# Bimetallic Single-Atom Nanozyme-Based Electrochemical-Photothermal Dual-Function Portable Immunoassay with Smartphone Imaging

**DOI:** 10.1021/acs.analchem.4c02606

**Published:** 2024-08-10

**Authors:** Yunsen Wang, Ruijin Zeng, Shuo Tian, Shuyun Chen, Zhilan Bi, Dianping Tang, Dietmar Knopp

**Affiliations:** † Key Laboratory of Analytical Science for Food Safety and Biology (MOE & Fujian Province), Department of Chemistry, Fuzhou University, Fuzhou 350108, People’s Republic of China; ‡ TUM School of Natural Sciences, Department of Chemistry, Chair of Analytical Chemistry and Water Chemistry, Technical University Munich, Lichtenbergstrasse 4, Garching 85748, Germany

## Abstract

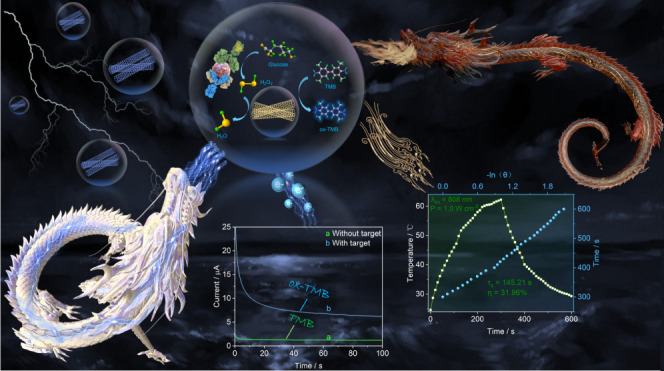

Rapid and accurate detection of human epidermal growth factor receptor 2 (HER2) is crucial for the early diagnosis and prognosis of breast cancer. In this study, we reported an iron–manganese ion N-doped carbon single-atom catalyst (FeMn-NC_etch_/SAC) bimetallic peroxidase mimetic enzyme with abundant active sites etched by H_2_O_2_ and further demonstrated unique advantages of single-atom bimetallic nanozymes in generating hydroxyl radicals by density functional theory (DFT) calculations. As a proof of concept, a portable device-dependent electrochemical-photothermal bifunctional immunoassay detection platform was designed to achieve reliable detection of HER2. In the enzyme-linked reaction, H_2_O_2_ was generated by substrate catalysis via secondary antibody-labeled glucose oxidase (GOx), while FeMn-NC_etch_/SAC nanozymes catalyzed the decomposition of H_2_O_2_ to form OH*, which catalyzed the conversion of 3,3′,5,5′-tetramethylbenzidine (TMB) to ox-TMB. The ox-TMB generation was converted from the colorimetric signals to electrical and photothermal signals by applied potential and laser irradiation, which could be employed for the quantitative detection of HER2. With the help of this bifunctional detection technology, HER2 was accurately detected in two ways: photothermally, with a linear scope of 0.01 to 2.0 ng mL^–1^ and a limit of detection (LOD) of 7.5 pg mL^–1^, and electrochemically, with a linear scope of 0.01 to 10 ng mL^–1^ at an LOD of 3.9 pg mL^–1^. By successfully avoiding environmental impacts, the bifunctional-based immunosensing strategy offers strong support for accurate clinical detection.

Breast cancer is the second most frequent cancer worldwide, and thus, timely screening of breast cancer is crucial in healthcare and treatment.^1,2^ Human epidermal growth factor receptor 2 (HER2) is a reliable, predictive, and prognostic biomarker for breast cancer due to the aberrant amplification of the HER2 driver gene, resulting in the overexpression of the HER2 protein and the formation of HER2-positive breast cancer. Of particular importance, HER2-positive breast cancer represents a significant fraction of breast cancers, with a prevalence of approximately 20–30%.^3,4^ Precise diagnosis of HER2 is crucial in selecting effective regimens for individualized treatment, given the rapid development of precision medicine. So far, various biomolecule detection and analysis methods have been developed using different signal transduction strategies, including electrochemical, photoelectrochemical, photothermal, and fluorescence analysis.^5,6^ Compared with analytical methods that require expensive instruments, photothermal and electrochemical analyses are cost-effective and offer high sensitivity, particularly making them widely used in health monitoring, environmental protection, and food analysis.^7,8^

Nanozymes are a class of functional nanomaterials with the ability to mimic the catalysis of natural enzymes, which are widely active in the fields of biomedicine and nanosensing due to their excellent multifunctionality.^9−12^ Various nanomaterials with enzyme-like activities, including noble metals, metal oxides, carbonyl nanomaterials, and metal–organic frameworks (MOF), have been developed by thoroughly analyzing the structure–effect relationships of nanozymes.^13−15^ The metal–nitrogen coordination structure (M–N-C) mimetic enzyme catalytic materials are designed using biomimicry with a catalytic center structure similar to that of natural enzymes, which confers a considerable catalytic capacity upon them.^16−19^ Regretfully, the restricted catalytic capacity of artificial nanozymes remains somewhat distant from that of naturally occurring enzymes, thus impeding the further utilization of nanozymes. Consequently, the logical development of highly active biomimetic nanozymes has emerged as a new area of study interest.^20−22^ Based on our latest research report, precise defect engineering and sensitive design of polymetallic active centers can effectively modify the local electronic structure and synergistically enhance the enzyme-mimetic catalytic activity of M–N-C nanomaterials, thereby improving the analytical performance of biosensing platforms.^23−27^

Electrochemical sensors are used by a broad spectrum of researchers their quick response, great sensitivity, and user-friendliness.^28−30^ Nevertheless, single-mode detection-based electrochemical test platforms cannot be guaranteed to be accurate, since they are vulnerable to outside interferences such as variations in measurement settings, operator preferences, and instrument variations. Dual signal transduction strategies, such as electrogenerated chemiluminescence (ECL)-electrochemistry or photoelectrochemistry-photothermal, have been developed recently to further improve the precision and accuracy of analytical strategies.^31−35^ Based on independent signals with different response mechanisms, the system error was calibrated by acquiring two independent signals and verifying them against each other. Photothermally responsive materials with acceptable stability are used in photothermal-based sensing platforms to relate photons, heat, and analytes.^36^ Due to their rich physicochemical properties, photothermal measurements also exhibit great potential for detection: (i) since photothermal signals originate from the photothermal effect, the environmental background has less of an impact on them; (ii) temperature-based detection technology requires only a light source and a thermometer to access the results; (iii) photothermal detection has a high signal-to-noise ratio and resistance to color interference compared to luminescence and colorimetric methods of visual analysis.^37,38^

Herein, we report iron–manganese-ion N-doped carbon single-atom catalysts (FeMn-NC_etch_/SAC) etched by H_2_O_2_, which enhance H_2_O_2_ adsorption by altering the electronic structure of the Fe sites, leading to a significant improvement in POD-like catalytic performance. FeMn-NC_etch_/SAC considerably enhanced the POD-like performance and raised its purity by 2.03 times as compared to Fe-NC/SAC, according to the enzyme viability test. Density functional theory (DFT) simulations show that FeMn-NC_etch_/SAC exhibits a lower energy barrier during the catalytic process, supporting the observed significant increase in the peroxidase activity. As a proof of concept, the bifunctional FeMn-NC_etch_/SAC nanozymes were employed as dual-signal recognition probes to catalyze the degradation of product H_2_O_2_, derived from glucose breakdown catalyzed by glucose oxidase. In turn, this caused TMB to oxidize, forming a blue oxide (ox-TMB) by an immune response mechanism that is unique to antigens and antibodies. Furthermore, the well-thought-out bioassay system, which relies on a laser (808 nm) and a microelectrochemical workstation, has minimal environmental requirements and does not need a special location for experimentation (Scheme 1). Owing to its mutually validated dual signal response mechanism and equipment portability, the model exhibits great competitiveness for developing additional portable detection platforms.

**Scheme 1:**
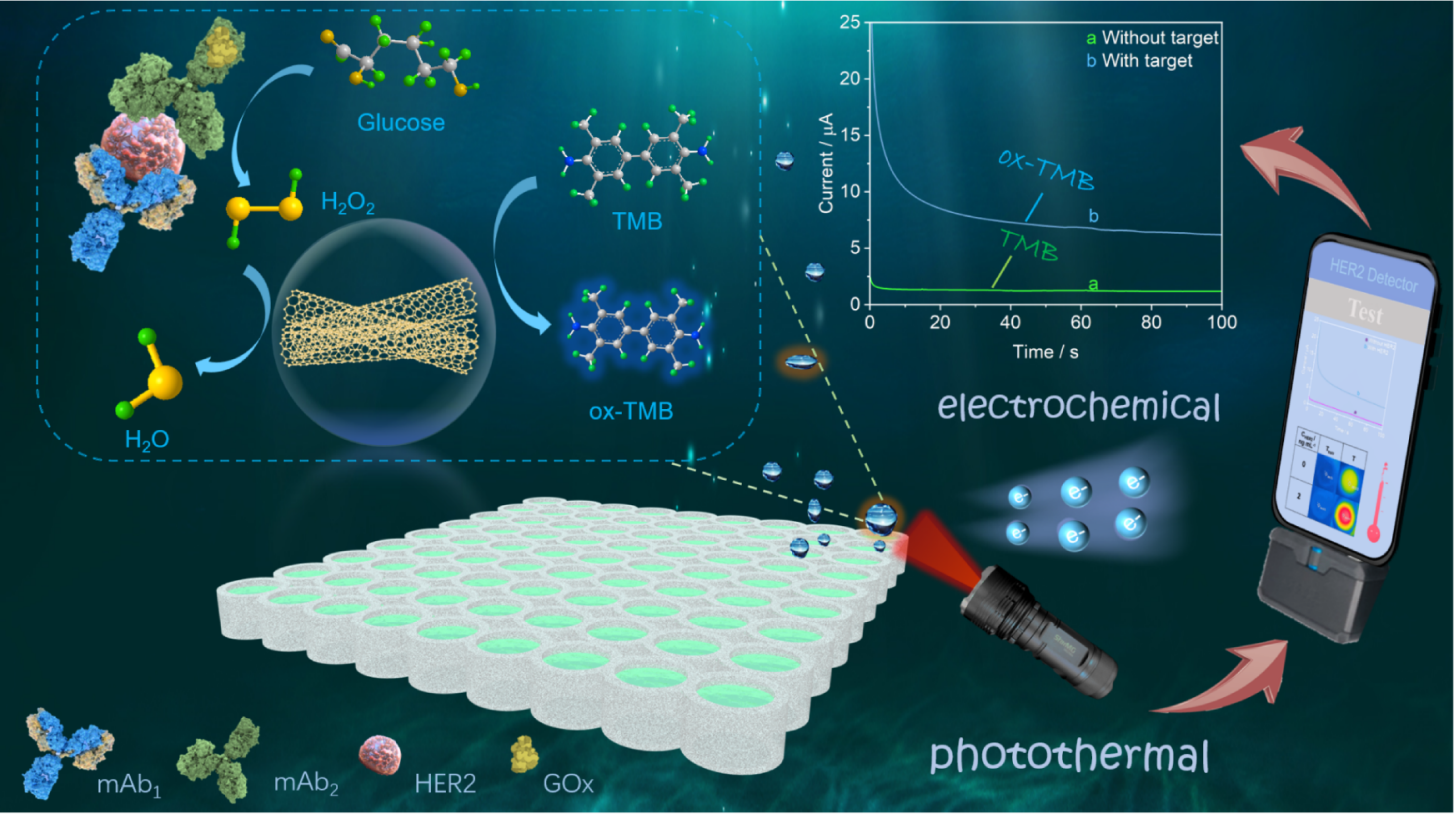
Schematic for Electrochemical-Photothermal Bifunctional Immunosensing of HER2 (mAb_1_: HER2 Capture Antibody; mAb_2_: Anti-HER2 Detection Antibody; GOx: Glucose Oxidase).

## Experimental Section

### Synthesis of ZnO Nanorods

In a typical synthesis of ZnO nanorods, NaOH (2.4 g) and zinc acetate dihydrate (0.66 g) were mixed in 5 mL of deionized water, including 20 mL of glycerol, under stirring. The mixture was kept in a Teflon-lined stainless steel autoclave at 150 °C for 24 h. The final product was treated with deionized water, centrifuged twice, and then vacuum-dried.

### Synthesis of Fe/Mn N-Doped Carbon Single-Atom Catalysts (FeMn-NC_etch_/SAC)

First, 100 mg of ZnO nanorods was dispersed by ultrasound in a solution containing 100 mg of 2-methylimidazole dissolved in 12 mL of dimethylformamide (DMF) and 4 mL of deionized water. ZnO@ZIF-8 nanorods were prepared through centrifugation, washing several times in ethanol and water, and then drying in a drying oven at 70 °C. Subsequently, the synthesized ZnO@ZIF-8 nanorods were heated for 2 h at 750 °C at a rate of 5 °C/min in an argon environment to obtain N-doped carbon (NC). The obtained NCs were dispersed in 1.0 M H_2_O_2_ solution, stirred for 1 h, and centrifuged. Then, FeCl_3_·6H_2_O (90 mg) and MnCl_2_·4H_2_O (66 mg) were added to prepare FeMn-NC_etch_/SAC, while FeCl_3_·6H_2_O (90 mg) was added to prepare Fe-NC_etch_/SAC. Lastly, these mixtures were stirred at 25 °C for 10 h and then dried at 100 °C. The dried powders were calcined at 800 °C under argon for 2 h.

### Peroxidase-like Activity of FeMn-NC_etch_/SAC

Generally, the peroxidase-like enzyme characteristics of FeMn-NC_etch_/SAC were examined using TMB during catalytic oxidation. First, 200 mM of NaAc–HAc solution (800 μL, pH 4.0) was mixed with 1.0 M of H_2_O_2_ solution (100 μL), 10 mM of TMB (100 μL), and 0.6 mg mL^–1^ of a FeMn-NC_etch_/SAC suspension (12 μL). Following a six-min period during which the blended solution was permitted to attain room temperature, the absorbance at 652 nm was quantified. Furthermore, by monitoring the absorbance of the TMB/H_2_O_2_/FeMn-NC_etch_/SAC system at 652 nm as a function of TMB and H_2_O_2_ concentrations, steady-state kinetic analysis of the TMB oxidation reaction was performed, which provided a clearer understanding of the peroxidase mimetic activity and the affinity relationship of FeMn-NC_etch_/SAC with TMB. From a Lineweaver–Burk plot, the Michaelis constant (*K*_m_) was obtained by employing the reciprocal of the Michaelis–Menten equation.

### Electrochemical and Photothermal Measurements of HER2

The immunoreactions were conducted in 96-well microtiter plates of high-binding polystyrene. Various concentrations of HER2 samples or 100 μL of standards were initially mixed with the mAb_1_-coated microtiter plates and incubated at 37 °C for 60 min. Then, the wells were washed with washing buffer, and a total of 100 μL of the mixture, GOx-labeled mAb_2_, was added and the mixture was incubated at 37 °C for 60 min. The microtiter plate was then washed as before. Then, 200 mM of NaAc–HAc solution (800 μL, pH 4.0), 10 mM of TMB (100 μL), and 0.6 mg mL^–1^ of FeMn-NC_etch_/SAC suspension (12 μL) were added to the wells, and the reaction was conducted for 20 min at 25 °C. After completing these procedures, electrochemical and photothermal measurements were performed. For electrochemical detection, the product was cast onto a clean screen-printed electrode for chronocurrent measurements. The initial voltage should be set to 0.1 V, with a sampling interval of 0.1 s and a run time of 100 s. The sensitivity should be set to 1.0 × 10^–5^ A/V. During photothermal detection, the temperature of the sample was monitored via a near-infrared imaging camera, while the reaction system was irradiated with an 808 nm laser at a power density of 1.0 W cm^–2^ for a cumulative irradiation time of 5 min. All experiments were conducted at 25 ± 1.0 °C.

## Results and Discussion

### Characterization of FeMn-NC_etch_/SAC

**Figure 1:**
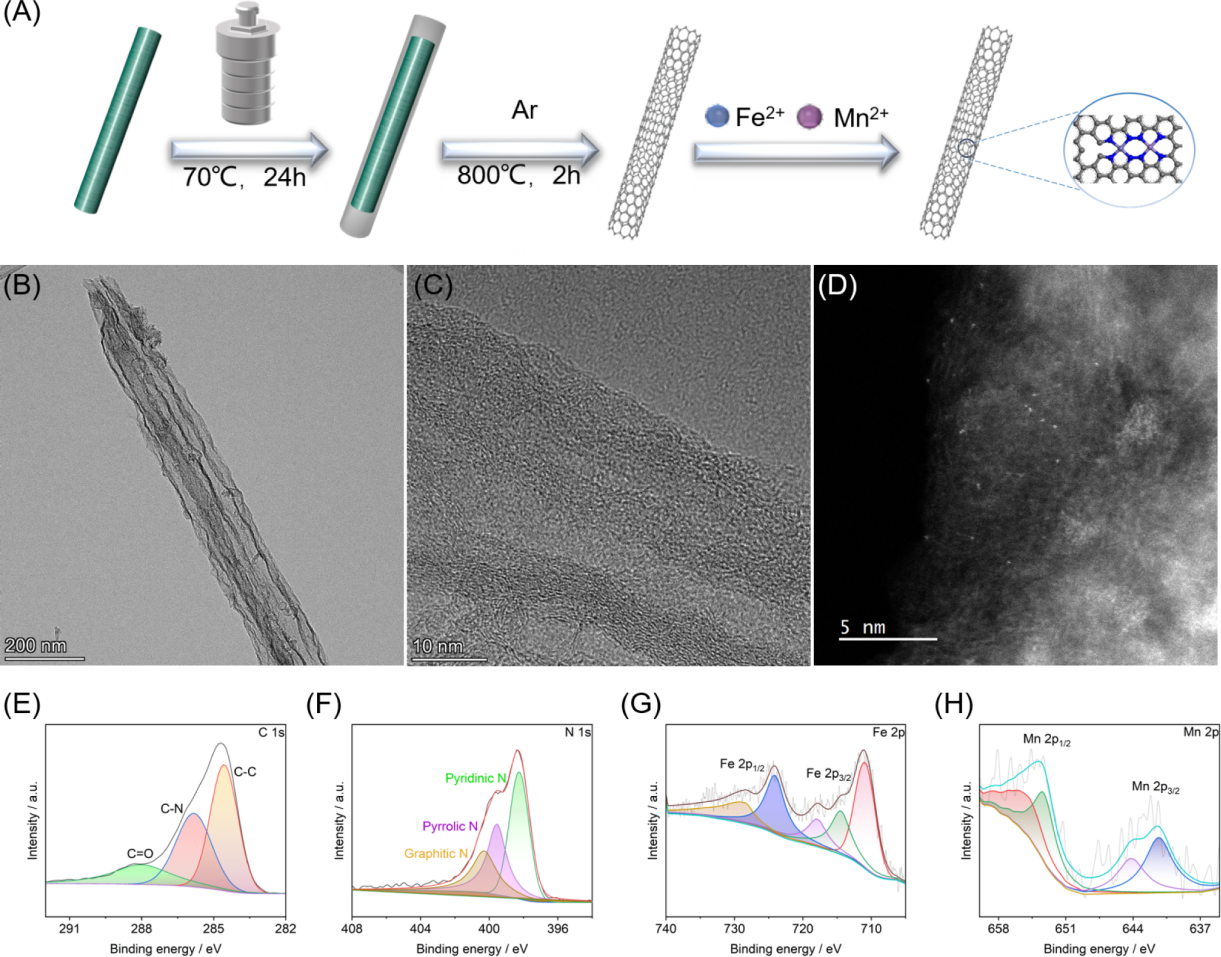
(A) Schematic diagram of FeMn-NC_etch_/SAC synthesis. (B) TEM image, (C) HRTEM image, and (D) aberration-corrected HAADF-STEM image of FeMn-NC_etch_/SAC. High-resolution XPS spectra of (E) C 1s, (F) N 1s, (G) Fe 2p, and (H) Mn 2p in FeMn-NC_etch_/SAC.

Fe–Mn bimetallic nitride-doped carbon was synthesized through a hydrothermal reaction and high-temperature annealing process utilizing a ZIF-8 template, as shown in Figure 1A. ZnO, NC precursors, and FeMn-NC_etch_/SAC were characterized by transmission electron microscopy (TEM). The results demonstrated that ZnO has a rod-like structure, whereas NC and FeMn-NC_etch_/SAC exhibit hollow tubular structures, as shown in Figures 1B and S1. The high-resolution transmission electron microscopy (HRTEM) images (Figure 1C) revealed that FeMn-NC_etch_/SAC exhibited the absence of metal nanoparticles/nanoclusters, indicating that there were no nanoparticles/nanoclusters attached to the surface of FeMn-NC_etch_/SAC. In addition, the presence of the Fe and Mn dual-single atoms is confirmed by the numerous bright spots that exhibit uniform dispersion on FeMn-NC_etch_/SAC in the aberration-corrected HAADF-STEM image, as shown in Figure 1D. Two peaks were discernible in the Raman spectrum of FeMn-NC_etch_/SAC at 1300 and 1600 cm^–1^, respectively. These peaks correspond to the D and G bands of the graphene structure, as illustrated in Figure S2. A rather large, low-intensity diffraction peak was observed during X-ray diffraction (XRD) examinations of the sample structure at 24.88°, which is attributed to the graphite-carbon (002) crystal plane (Figure S3).

Furthermore, an X-ray photoelectron spectroscopy (XPS) measurement was conducted to more accurately ascertain the valence states and composition of FeMn-NC_etch_/SAC. In the C 1s spectra, the C–C, C–N, and C=O exhibit three distinct single peaks at 284.7, 285.8, and 288.1 eV, respectively (Figure 1E). In the XPS high-resolution spectra of the N 1s, the pyridine, pyrrole, and graphitic nitrogen yield three distinct peaks at 398.4, 399.6, and 400.3 eV, respectively, as shown in Figure 1F. In the interim, two double peaks were observed in the high-resolution spectra of Fe 2p, with binding energies of 711.4 and 728.5 eV. Similarly, two double peaks were seen in the high-resolution spectra of Mn 2p, with binding energies of 654.6 and 641.4 eV. In the distinctive spin–orbital splitting of divalent manganese and iron at Fe 2p_3/2_, Fe 2p_1/2_, and Mn 2p_3/2_, Mn 2p_1/2_ is responsible for the observed peaks (Figure 1G,H).

### Density Functional Theory (DFT) Simulation

**Figure 2:**
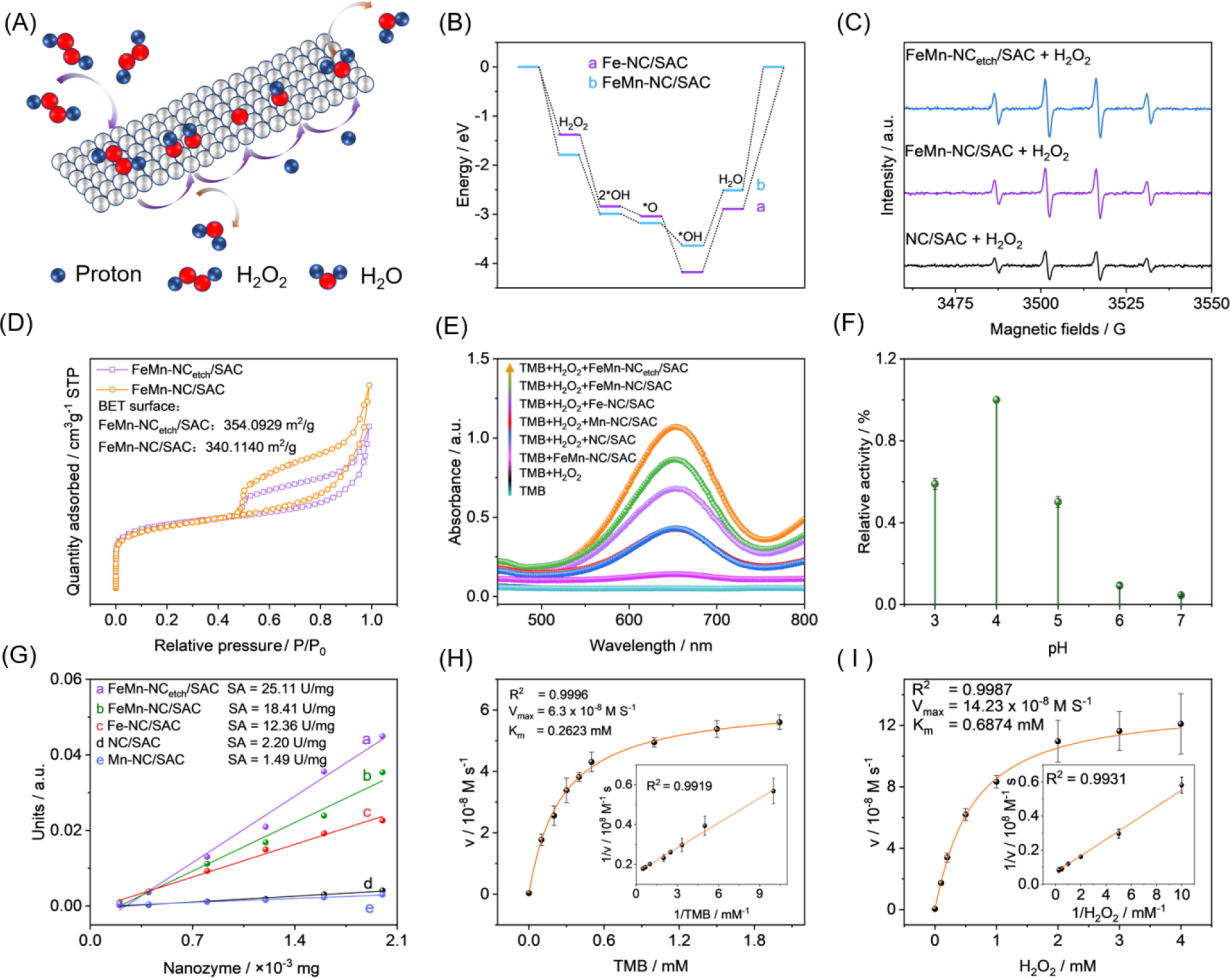
(A) Schematic illustration of H_2_O_2_ decomposition catalyzed by FeMn-NC/SAC. (B) The free-energy diagrams of the POD-like mechanisms on FeMn-NC/SAC. (C) EPR spectra of OH* trapped by DMPO in FeMn-NC_etch_/SAC + H_2_O_2_, FeMn-NC/SAC + H_2_O_2_, and NC/SAC + H_2_O_2_. (D) Brunauer–Emmett–Teller isotherms of FeMn-NC_etch_/SAC and FeMn-NC/SAC. (E) UV–vis spectra of different solutes. (F) Relative activity plots for different pH values. (G) Specific activity of FeMn-NC_etch_/SAC, FeMn-NC/SAC, Fe-CN/SAC, Mn-CN/SAC, and NC/SAC. FeMn-NC_etch_/SAC nanozyme Michaelis–Menten kinetics and pertinent double reciprocal graphs using (H) TMB and (I) H_2_O_2_ as the substrates.

It is typical for FeMn-NC/SAC to catalyze the decomposition of H_2_O_2_ and oxidize colorless TMB to blue ox-TMB via intermediate products. DFT calculations were performed on the FeMn-NC/SAC model and Fe-CN/SAC to further clarify the exceptional performance of bimetallic atom doping. The catalytic mechanism process is shown in Figure 2A. The degradation behavior of hydrogen peroxide at the bimetallic single-atom catalytic interface is thought to be related to the decomposition of H_2_O_2_ at the interface, two-step protonation of the O atoms, and desorption of water. As illustrated in the Gibbs free energy diagram (Figure 2B), the H_2_O_2_ molecule is uniformly cleaved at the active site of the Fe monatomic to produce two OH*, and the reactions at the OH* formation step are all exothermic and exhibit thermodynamic spontaneity. Under acidic conditions, O* was generated as OH* at the monatomic Mn site. Notably, the rate-determining step (RDS) is a surface hydrolysis adsorption process, namely, OH* + H^+^ + H_2_O → 2H_2_O. In contrast, the energy barrier of FeMn-NC/SAC (1.13 eV) is lower than that of Fe-CN/SAC (1.29 eV), which results in FeMn-NC/SAC, exhibiting superior peroxide-like activity. The FeMn-NC/SAC-catalyzed H_2_O_2_ decomposition mechanism is as follows:
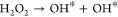

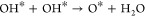

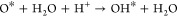

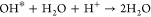


### Peroxidase-like Activity of FeMn-NC_etch_/SAC

The intermediates of the catalytic process in Figure 2C were investigated using electron paramagnetic resonance (EPR) to elucidate the catalytic mechanism of FeMn-NC_etch_/SAC. FeMn-NC_etch_/SAC was combined with 5,5-dimethyl-1-pyrroline-*N*-oxide (DMPO, 100 mM) acting as a trapping agent, and hydrogen peroxide (H_2_O_2_, 100 mM, 60 μL) was used as the substrate. As illustrated, after 5 min, the EPR spectra of FeMn-NC_etch_/SAC, FeMn-NC/SAC, and CN/SAC exhibited distinct quadruple characteristic peaks with an intensity ratio of 1:2:2:1. Of greater significance, the FeMn-NC_etch_/SAC system exhibited a higher absorbance than the FeMn-NC/SAC and CN/SAC systems, demonstrating that FeMn-NC_etch_/SAC had a stronger OH* capacity. Subsequently, the effect of H_2_O_2_ etching on the specific surface area of FeMn-NC_etch_/SAC was evaluated by conducting a Brunauer–Emmett–Teller (BET) curve analysis. As illustrated in Figure 2D, the curves demonstrated that the specific surface area of FeMn-NC/SAC was 340.11 m^2^/g before H_2_O_2_ etching and increased to 354.09 m^2^/g after H_2_O_2_ etching. This indicated that H_2_O_2_ etching resulted in the formation of more porous structures. The H_2_O_2_ etching process created numerous pores, with an average pore size of 1.74 nm (Figure S4). The enhanced catalytic properties of the pores were evidenced by their capacity to accommodate a greater number of metal atoms.

To demonstrate the peroxide-like ability of FeMn-NC_etch_/SAC, control experiments were conducted using TMB as a chromogenic substrate. A colorimetric system of TMB, H_2_O_2_ solution, and nanozymes (FeMn-NC_etch_/SAC, FeMn-NC/SAC, Fe-CN/SAC, Mn-CN/SAC, and CN/SAC) was constructed using the TMB solution and TMB + H_2_O_2_ solution as control, and the UV absorption of the system at 652 nm was tested. Figure 2E illustrates that the mixture comprising TMB, H_2_O_2_, and nanozymes exhibited a notable absorption at 652 nm in comparison to the control, where FeMn-NC_etch_/SAC exhibited the highest absorbance, confirming that FeMn-NC_etch_/SAC exhibits significant peroxidative activity. Furthermore, the effect of pH on the system was considered, and a sodium acetate buffer solution with a pH range of 3.0 to 7.0 was prepared to study the impact of the environment’s acidity on the catalytic activity of the nanozymes. The results of the experiments demonstrated that the nanozymes exhibited their maximum catalytic activity at pH 4.0 (Figure 2F).

In addition, the specific activity of each nanozyme was quantified and showed that FeMn-NC_etch_/SAC (25.11 U/mg) had a significantly higher specific activity than did the other nanozymes of the same group (Figure 2G). By adjustment of the quantities of H_2_O_2_ and TMB, the apparent steady-state kinetic characteristics of the TMB/H_2_O_2_/FeMn-NC_etch_/SAC system were quantified, allowing for a more precise determination of the catalytic efficiency of FeMn-NC_etch_/SAC. Both the Lineweaver–Burk double inverse model and the Michaelis–Menten model were used to fit the experimental data, with consistent outcomes. Key steady-state kinetic parameters, such as the Michaelis constant (*K*_m_) and maximal response rate (*V*_max_), were computed based on the slopes and intercepts found from Lineweaver–Burk double inverse plots (Figure 2H,I). Notably, the *K*_m_ of TMB was determined to be 0.26 mM, while *V*_max_ was found to be 6.3 × 10^–8^ Ms^–1^. Furthermore, varying H_2_O_2_ concentration in steady-state kinetic experiments gave a *K*_m_ value of 0.69 mM and a *V*_max_ value of 14.23 × 10^–8^ Ms^–1^, demonstrating the remarkable stability of nanozymes toward the substrate. As shown in Table S1, the FeMn-NC_etch_/SAC nanozyme had a lower *K*_m_ and a higher *V*_max_ than many other nanozymes, indicating a stronger affinity for TMB substrates and superior catalytic activity.

### Feasibility Assessment of the Bifunctional Sensing Platform

Building on the previous discussion, it was demonstrated that FeMn-NC_etch_/SAC has highly proficient peroxide-like activity. This, in turn, facilitated the development of a dual readout biosensor with photothermal and electrochemical functionalities for the immunosensing detection of HER2. The immobilization of HER2 was achieved using a sandwich structure as a template, where glucose oxidase (GOx) was immobilized on a secondary antibody and catalyzed the production of H_2_O_2_ from substrate glucose. FeMn-NC_etch_/SAC then catalyzed H_2_O_2_ and oxidized TMB to obtain the electroactive substance ox-TMB. This process achieved both photothermal and electrical signal outputs, indirectly enabling the process of HER2 quantification.

**Figure 3:**
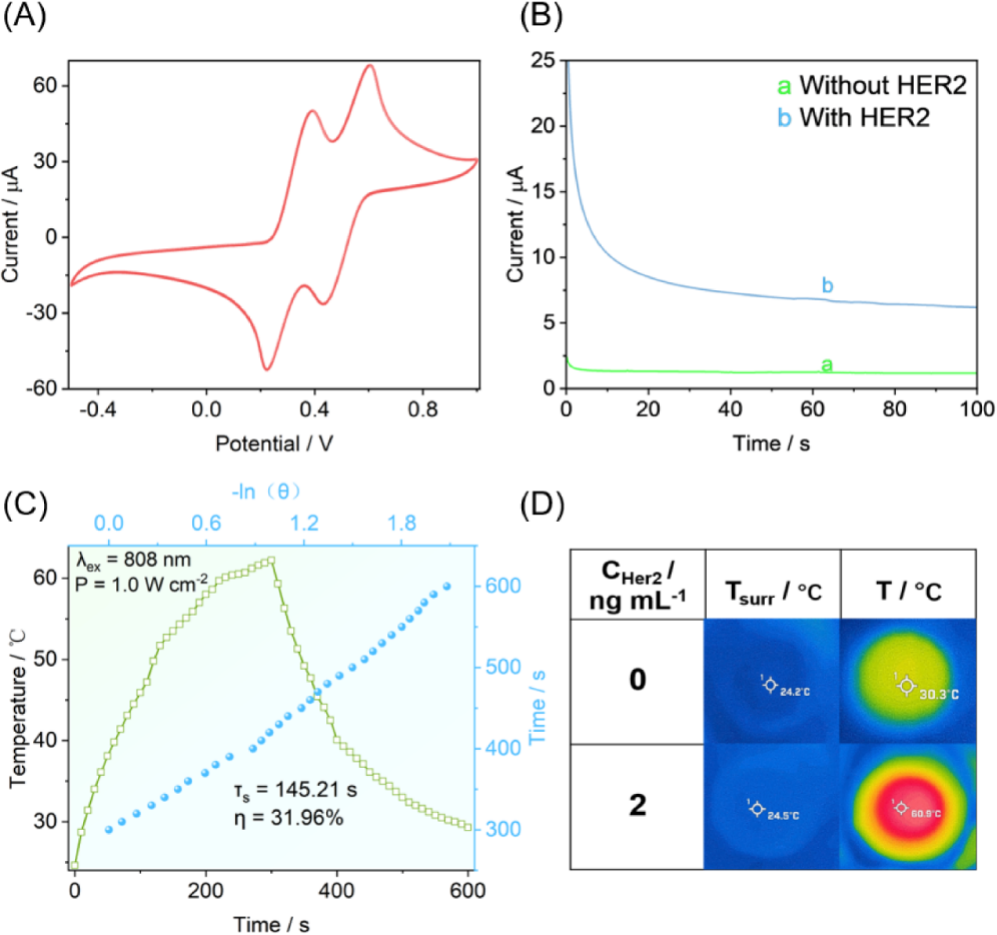
(A) CV of TMB. (B) Feasibility of electrochemical detection. (C) Heating and cooling curves of an FeMn-NC_etch_/SAC aqueous solution under 808 nm laser irradiation. (D) Feasibility of photothermal detection.

In light of the potential for the electrocatalytic process and the photothermal protocol of TMB, cyclic voltammetry (CV) and calculation of the photothermal efficiency were applied to the TMB solution as shown in Figure 3A. In the absence of exogenous substances, the CV curves of TMB exhibited significant redox peaks corresponding to two-electron redox reactions, indicating that the electronic communication at the sensing interface was unobstructed and that ox-TMB could be reduced by applying a certain bias voltage. Meanwhile, the photothermal conversion efficiency was determined using the photothermal heating curves of the pure TMB solution and the FeMn-NC_etch_/SAC reaction system. Surprisingly, the reaction system exhibited a photothermal conversion efficiency (31.96%) that was much higher than that of the pure TMB solution (6.29%) (Figures 3C and S5), demonstrating the dominance of the photothermal sensors.

Furthermore, a digital thermal infrared imager and a portable miniature electrochemical workstation were employed as signal output platforms to achieve portable HER2 detection (note: the detection devices are illustrated in Figures S6 and S7). These portable devices were utilized to record the chronocurrent and temperature signals with and without HER2. In the absence of HER2, no discernible electrical signal was observed from the chronocurrent, whereas in the presence of HER2, a significant electrical response signal was detected from the sensor as shown in Figure 3B. Similarly, in the absence of HER2, the photothermal signal change was comparable to that of the pure TMB solution, and the temperature response was significantly enhanced by the addition of HER2 as shown in Figure 3D. To assess the stability of thermal images with a mobile phone, a 0.2 ng mL^–1^ of HER2 sample was incubated for photothermal imaging stability testing, as detailed in the Photothermal Measurement Methods and in Figure S8. The average temperature increase was 26.84 °C over nine replicated trials with a dynamic range of 26.6–27.1 °C. The standard deviation (SD) was calculated as 0.45, with a relative standard deviation (RSD) of 1.69%. The experimental results showed that the photothermometric imaging approach was reliable and the results were stable. This suggests that successfully constructing a dual-signal transmission sensor offers the accurate detection of HER2 in the clinical setting.

### Anti-Tumor Therapeutic Potential of FeMn-NC_etch_/SAC

**Figure 4:**
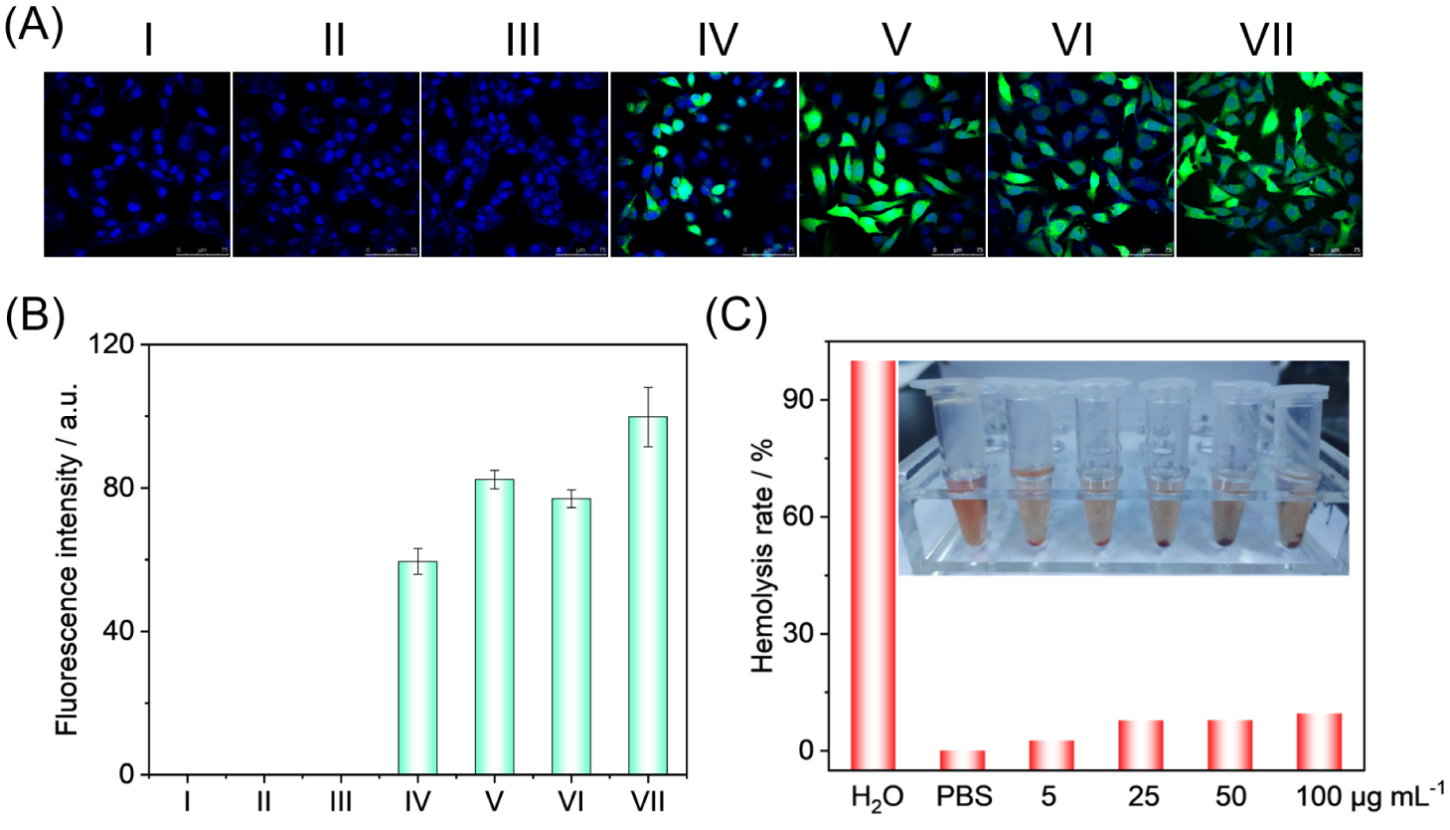
Fluorogram (A) and fluorescence intensity (B) of 4T1 cells labeled with DCFH-DA in (I) phosphate-buffered saline (PBS), (II) NIR, (III) H_2_O_2_, (IV) FeMn-NC_etch_/SAC, (V) FeMn-NC_etch_/SAC + NIR, (VI) FeMn-NC_etch_/SAC + H_2_O_2_, and (VII) FeMn-NC_etch_/SAC + H_2_O_2_ + NIR. (C) Hemolytic analysis of erythrocytes after incubation with different concentrations of FeMn-NC_etch_/SAC (5, 25, 50, and 100 μg mL^–1^).

Considering the excellent enzyme-like activity and efficient photothermal properties of FeMn-NC_etch_/SAC, its potential therapeutic efficacy in tumor therapy was further evaluated. The potential mechanism of 4T1 cell death was investigated by employing 2,7-dichlorodihydrofluorescein diacetate (DCFH-DA) as a sensitive indicator, which can undergo oxidation by intracellular reactive oxygen species (ROS) to generate the green fluorescent compound 2′,7′-dichlorofluorescein (DCF). Negligible green fluorescence was observed in the phosphate-buffered saline (PBS) group, the near-infrared (NIR) group, and the hydrogen peroxide (H_2_O_2_) group. This suggests that there was no intracellular ROS generation in the aforementioned treatments. In contrast, intracellular ROS were generated in the FeMn-NC_etch_/SAC group, FeMn-NC_etch_/SAC + NIR group, FeMn-NC_etch_/SAC + H_2_O_2_ group, and FeMn-NC_etch_/SAC + NIR + H_2_O_2_ group, which exhibited green fluorescence in Figure 4A,B. Notably, the FeMn-NC_etch_/SAC + H_2_O_2_ + NIR group exhibited the strongest green fluorescence, which was attributed to the synergistic effect of photothermal catalysis in enhancing intracellular ROS generation. Furthermore, the results of hemolysis of mouse erythrocytes demonstrated that FeMn-NC_etch_/SAC has good in vivo biocompatibility, with a hemolysis rate of less than 10% in various concentrations of FeMn-NC_etch_/SAC (Figure 4C).

### Validation of Electrochemical-Photothermal Sensor for the Detection of HER2

**Figure 5:**
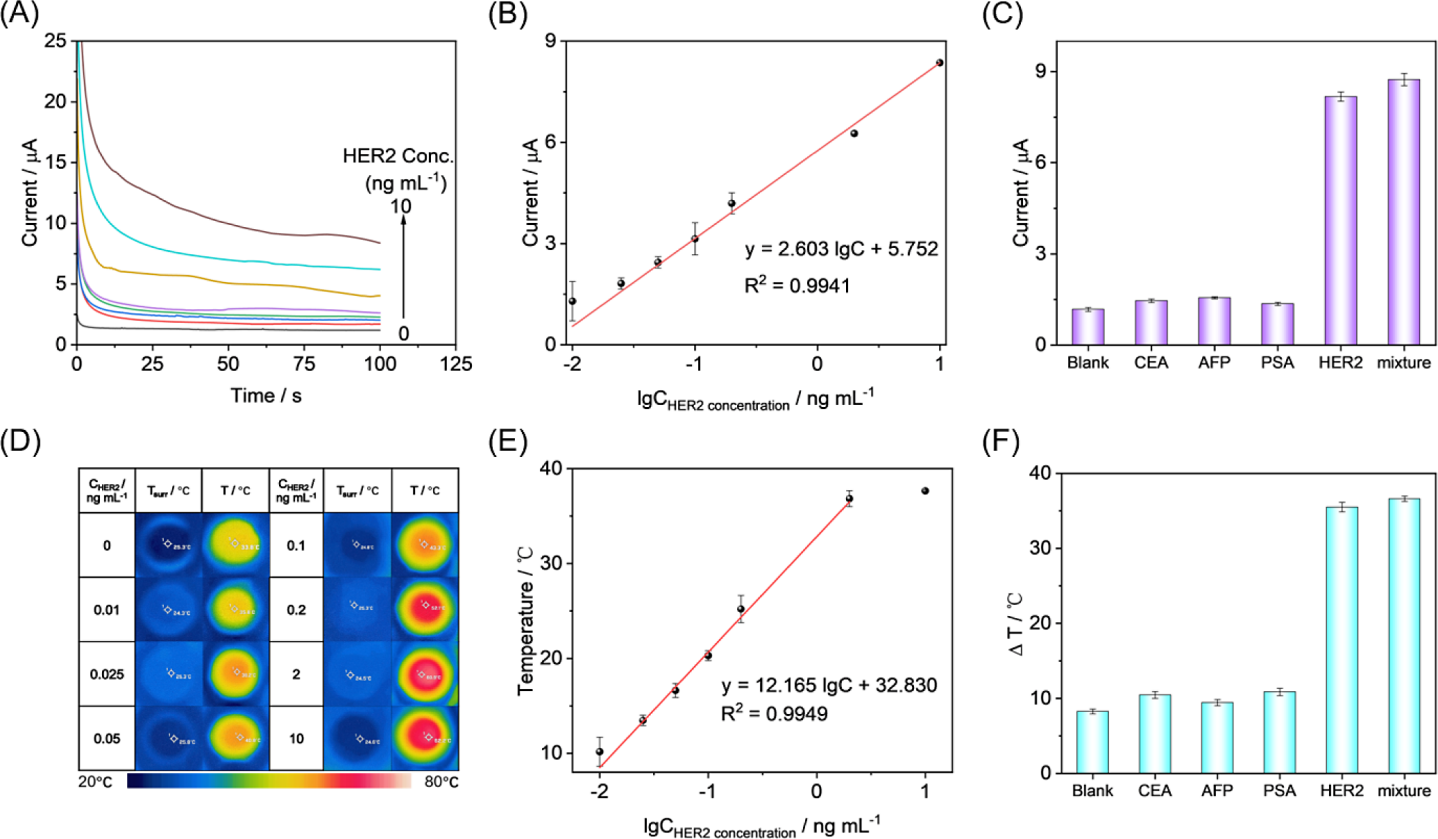
(A) Time-current curve toward the HER2 concentration from 0.01 to 10 ng mL^–1^. (B) Linear relationship between *I* and HER2 concentration. (D) Photomicrographs of the photothermal detection system before and after irradiation for different HER2 concentrations (0.01–10 ng mL^–1^). (E) linear relationship between Δ*T* and HER2 concentration. Selectivity of the (C) electrochemical and (F) photothermal immunoassay detection.

The sensor’s performance in detecting HER2 was evaluated by creating a series of concentration gradients based on the successful development of a bifunctional immunosensor. The test current value of the sensor increased linearly withthe increasing HER2 concentration over a range of 0.01–10 ng mL^–1^ (Figure 5A). The response current values were collected at 100 s for different concentrations of HER2, and a good linear relationship was found between the HER2 concentration and the test current. The data were fitted to yield a linear equation of *I* = 2.603 lg of *C* + 5.752 (*n* = 7, *R*^2^ = 0.9941), and the limit of detection (LOD) was 3.9 pg mL^–1^ at S/N = 3 (Figure 5B). This result was superior to that of the existing HER2 detection strategy (Table S2). Meanwhile, the photothermal sensing performance was carried out at this concentration gradient. The temperature difference was recorded over 6 min using 808 nm infrared laser irradiation, and the data were analyzed linearly against the HER2 concentration. This analysis revealed a good linear relationship between the HER2 concentration and the recorded temperature difference in the 0.01–2 ng mL-1 range as shown in Figure 5D. The data were fitted to yield a linear equation of Δ*T* = 12.165 lg *C* + 32.830 (*n* = 6, *R*^2^ = 0.9949, and Δ*T* = *T* – *T*_surr_), with a calculated LOD of 7.5 pg mL^–1^ at S/N = 3 (Figure 5E). Compared with the two methods, electrochemical detection has the advantages of high sensitivity, low detectability, and wide detection range, while photothermal detection has a stronger anti-interference ability.

A bifunctional sensing approach combining electrochemical and photothermal techniques was employed to investigate the specificity of the HER2 detection method. Alpha-fetoprotein (AFP), prostate-specific antigen (PSA), and carcinoembryonic antigen (CEA) at a concentration of 10 ng mL^–1^ were utilized as potential interfering agents in controlled experiments. The results of the experiments are presented in Figure 5C,F. The current and photothermal responses to the interfering substances were minimal compared to the empty control group. However, in the presence of the HER2 target molecule, the experimental and mixed experimental groups exhibited considerable current and photothermal responses. These results demonstrate that the constructed electrochemical-photothermal bifunctional immunosensor has good anti-interference ability.

### Preliminary Application of Real Samples

To evaluate the viability of this bifunctional sensing platform for clinical diagnostic applications, five human serum samples were obtained from Mengchao Hepatobiliary Hospital in Fuzhou, China, where samples were monitored using this method and the commercial HER2 ELISA kit (as a reference). The Supporting Information provides detailed instructions on preparing human serum samples before measurements. The results are presented in Table 1, indicating no significant difference between the mean values of the dual-readout test and those of a commercially available HER2 ELISA kit. All experimental values (*t*_exp_) were found to be below the critical value (*t*_crit_) of 2.78 (*t*_crit [0.05,4]_ = 2.78), thereby demonstrating the satisfactory accuracy of the dual-readout sensors. Consequently, it can be concluded that the designed dual-readout biosensor holds promising potential for accurately detecting HER2 in practical applications.

**Table 1:** Detection of HER2 in Human Serum Samples Using the Electrochemical Immunoassay and a Commercial HER2 ELISA Kit

	method; concn (mean ± SD, ng mL^–1^, *n* = 3)				
sample no.	electrochemical immunoassay	RSD (%)	HER2 ELISA kit	RSD (%)	*t*_exp_
1	0.32 ± 0.02	6.25	0.34 ± 0.03	8.82	0.96
2	4.59 ± 0.21	4.58	4.33 ± 0.18	4.16	1.62
3	9.86 ± 0.30	3.04	9.67 ± 0.25	2.59	0.84
4	1.36 ± 0.08	5.88	1.29 ± 0.06	4.65	1.21
5	8.02 ± 0.20	2.49	7.88 ± 0.17	2.16	0.92

## Conclusions

In conclusion, we have designed an electrochemical and photothermal bifunctional HER2 detection platform based on the FeMn-NC_etch_/SAC nanozyme. The designed innovative platform has the following advantages: (i) the construction of defective active sites allows for effective doping of bimetallic atoms, significantly enhancing the POD-like activity of the nanozymes; (ii) the bioanalyzer, based on a portable laser (808 nm), a digital multimeter, and a miniature electrochemical workstation, has two independent output signals for more accurate and reliable results and has great potential for point-of-care detection; (iii) the material is well-biocompatible and has the potential for this entity to be developed into a biotherapeutic agent. This study of the sensing platform, which can achieve satisfactory detection performance without laborious modification of the electrode surface, offers a comprehensive foundation for accurate early tumor diagnosis and prediction.

## Supporting Information Available

Reagents and materials, instrumentation, preparation of human serum specimens, commercial ELISA for HER2, Figures S1–S8, and Tables S1 and S2 (PDF)
