# Supplementary material for: Bimetallic Single-Atom Nanozyme-Based Electrochemical-Photothermal Dual-Function Portable Immunoassay with Smartphone Imaging
Source: Anal Chem. 2024 Aug 10;96(33):13663–71. doi: 10.1021/acs.analchem.4c02606 (PMC11339724; doi:10.1021/acs.analchem.4c02606)
Supplement: Supplementary file 1 — ac4c02606_si_002.pdf [file ac4c02606_si_002.pdf]

SUPPORTING INFORMATION

Bimetallic Single-Atom Nanozyme-Based Electrochemical-Photothermal Dual-Function Portable Immunoassay with Smartphone Imaging

Yunsen Wang,[†] Ruijin Zeng,[†] Shuo Tian,[†] Shuyun Chen,[†] Zhilan Bi,[†] Dianping Tang,[†,*] and Dietmar Knopp[ξ,*]

[†] Key Laboratory of Analytical Science for Food Safety and Biology (MOE & Fujian Province), Department of Chemistry, Fuzhou University, Fuzhou 350108, People's Republic of China

[ξ] Technical University Munich, TUM School of Natural Sciences, Department of Chemistry, Chair of Analytical Chemistry and Water Chemistry, Lichtenbergstrasse 4, 85748 Garching, Germany

*Corresponding Authors: Phone: +86-591-2286 6125; fax: +86-591-2286 6135; e-mails: dianping.tang@fzu.edu.cn (D. Tang); dietmar.knopp@mytum.de (D. Knopp)

TABLE OF CONTENTS

Reagents and materials...S**Error! Bookmark not defined.**

Instruments...S**Error! Bookmark not defined.**

Preparation of human serum specimens...S**Error! Bookmark not defined.**

Commercial ELISA for HER2 ....S4

Figure S1 ....S5

Figure S2 ....S5

Figure S3 ....S6

Figure S4 ....S6

Figure S5 ....S7

Figure S6 ....S8

Figure S7 ....S9

Figure S8 ....S10

Table S1 ....S11

Table S2 ....S12

REFERENCES S13

Reagents and Materials. All chemicals or reagents were obtained from commercial suppliers and used as received with an analytical grade. Sodium hydroxide (beads, 97.0%), glycerine (AR,99%), acetic acid, ethanol (99.9%, anhydrous), zinc acetate ($Zn(CH_3COO)_2$), 2-methylimidazole ($C_4H_6N_2$), iron (III) chloride hexahydrate ($FeCl_3{\cdot}6H_2O$), manganese (II) chloride tetrahydrate ($MnCl_2{\cdot}4H_2O$), 5,5-dimethyl-1-pyrroline-N-oxide (DMPO), 2,7-dichlorodihydrofluorescein diacetate (DCFH-DA), hydrogen peroxide (H_2O_2, 30wt% in H_2O), 3,3′,5,5′-tetramethylbenzidine (TMB) and N, N-dimethylformamide (DMF) were obtained from Aladdin (Shanghai, China). The ultrapure water used in all solutions was obtained from a Millipore Milli-Q water purification system (Billerica, MA) with a resistivity of 18.25 MΩ·cm. Screen-printed carbon electrodes were purchased from Taobao Electrochemical Sensing Shop (4 mm diameter). Human epidermal growth factor receptor 2 (HER2) ELISA kits were obtained from Wuhan Cusabio Biotech. Inc. (Wuhan, China).

Instruments. Transmission electron microscopy (TEM) images were obtained on a Thermo Scientific Talos F200i instrument. X-ray photoelectron spectra (XPS) were obtained from a Thermo Scientific K-Alpha X-ray photoelectron spectrometer with an excitation source of monochromatized Al Ka (hv = 1486.6 eV). The adventitious C 1s peak was set at 284.8 eV for charge correction purposes. The powder X-ray diffraction (PXRD) patterns were recorded by a Rigaku Smartlab SE X-ray diffractometer with Cu Kα lines. The UV-vis diffuse reflectance spectra (UV-vis DRS) were recorded on a Shimadzu UV-3600 Plus UV-vis-NIR spectrophotometer. A CHI 850D electrochemical workstation and a three-electrode system consisting of a GCE/sample as the working electrode, a Pt wire as the counter electrode, and an Ag/AgCl electrode as the reference electrode were used to record time-currents date (i-t curves).

Preparation of Human Serum Specimens. Before measurement, these collected human serum samples from Fujian Provincial Hospital (Fuzhou, China) were initially centrifuged for 5 min at 4 °C with 5,000*g* (centrifugal force) to remove the possibly existing impurities and macromolecules. Thereafter, the obtained supernatant fluids were determined using the developed immunoassay and the commercialized human HER2 ELISA kit, respectively. All participants in this experimental experiment provided informed consent before the experiment. All the experiments were performed in accordance with the Guidelines of Fuzhou University (China), and approved by the ethics committee at Fuzhou University (China).

Commercial ELISA for HER2. A commercially available ELISA assay was utilized for method comparison studies. In sandwiched ELISA with standard polystyrene 96–well plates, 50 μL serum sample suspension was incubated at 37 °C for 30 min, and the wells were rinsed 3 times (3 min each) with 0.1 mol L^{-1} PBS (pH 7.4) containing 0.5 mol L^{-1} NaCl and 1.0 mg mL^{-1} Tween 20. Then we added 50 μL conjugate solution and incubation continued for 60 min. The wells were again rinsed and 50 μL 3,3',5,5'-tetramethylbenzidine (TMB) reagent was added and incubated at 37 °C for 10 min. The enzymatic reaction was stopped by adding 50 μL of 2.0 mol L^{-1} H_2SO_4 to each well. The results of ELISA were measured by a spectrophotometric ELISA reader at a wavelength of 450 nm.

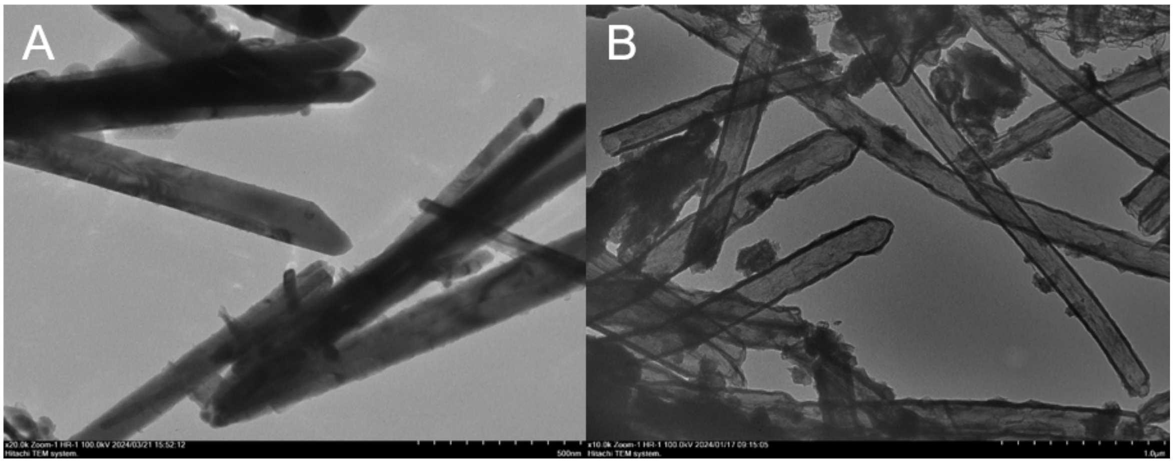

Figure S1. TEM image of (A) ZnO, and (B) NC.

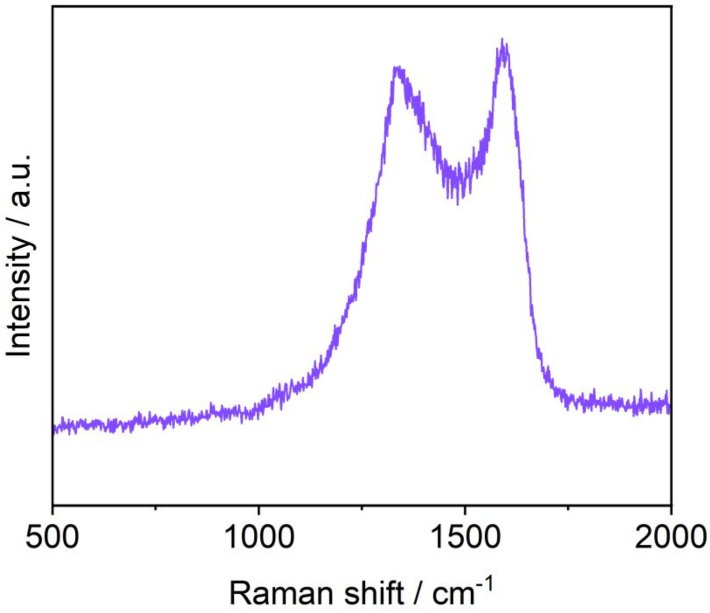

Figure S2. Raman spectrum of FeMn-NC_{etch}/SAC.

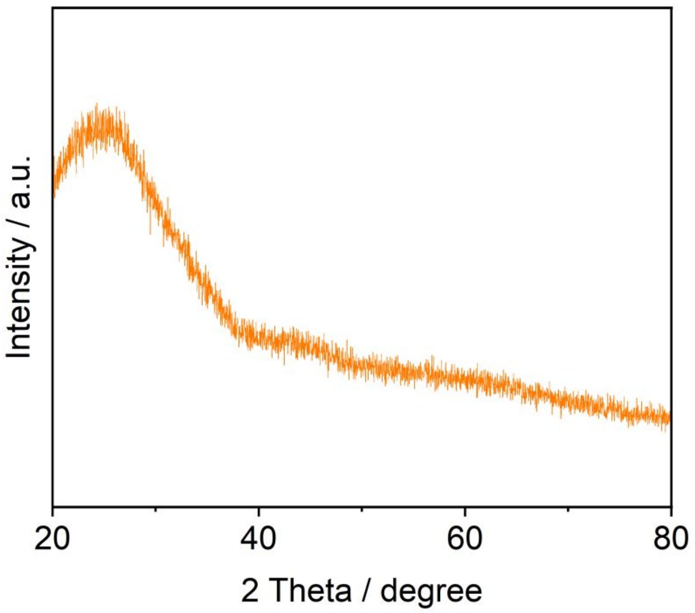

Figure S3. XRD pattern of FeMn-NC_{etch}/SAC.

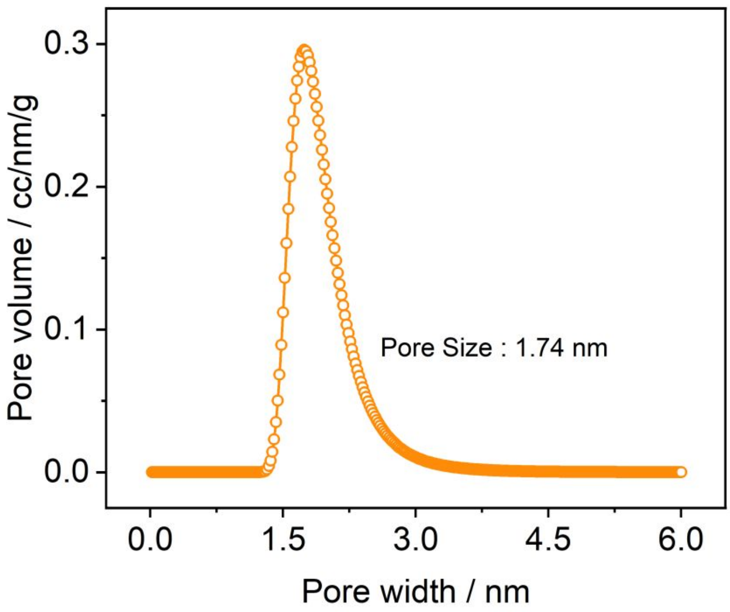

Figure S4. The pore width distribution of FeMn-NC_{etch}/SAC.

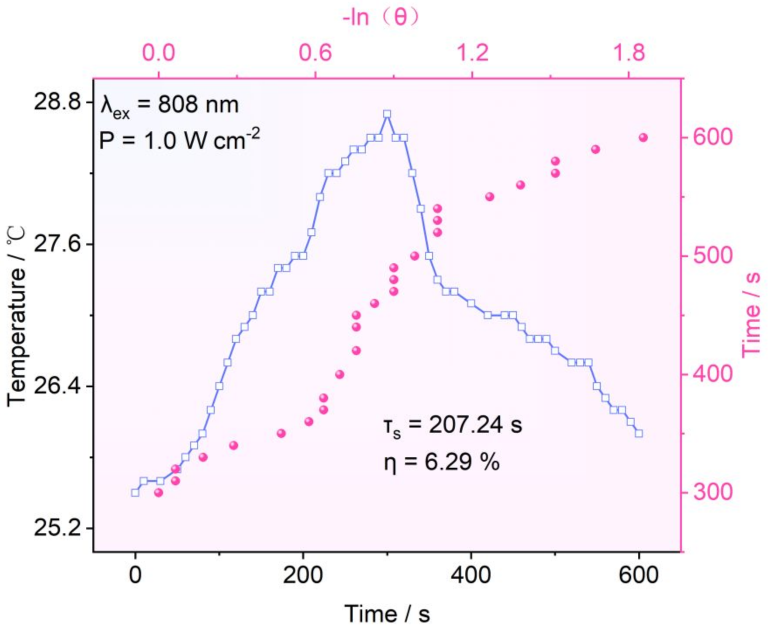

Figure S5. Heating curves under 808 nm laser irradiation (P = 1.0 W) and linear regression of cooling time and minus natural logarithm of driving force temperature of TMB.

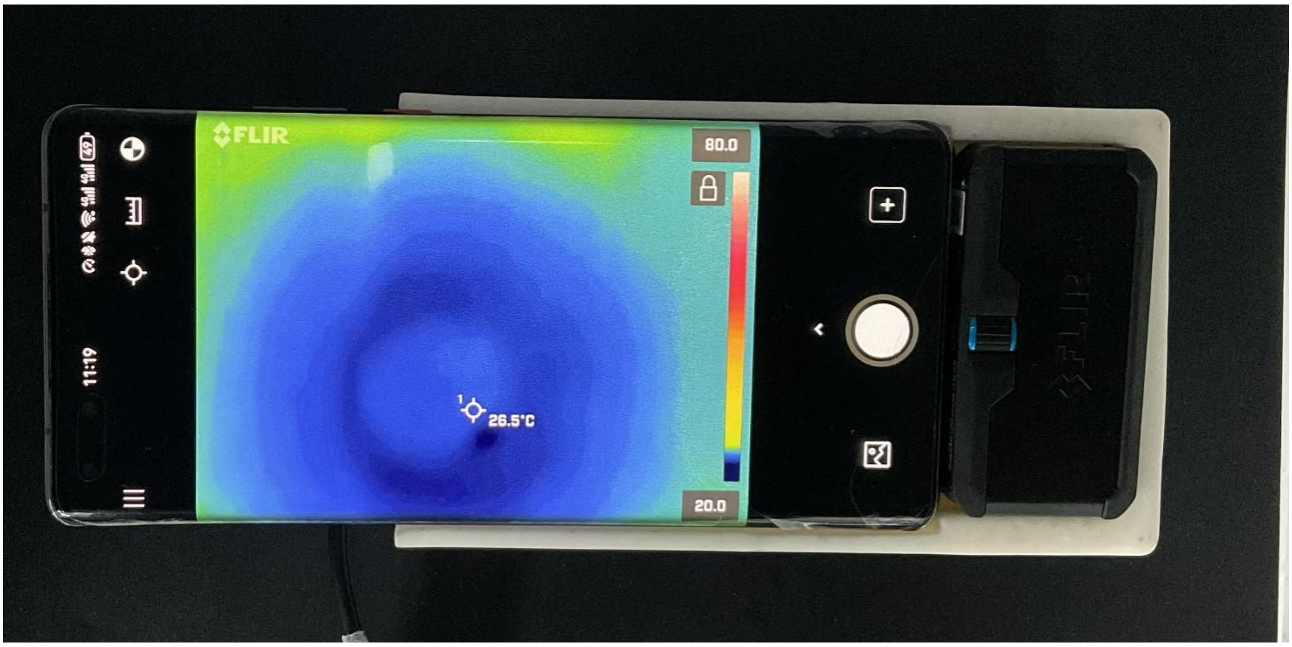

Figure S6. The portable photothermal detection device.

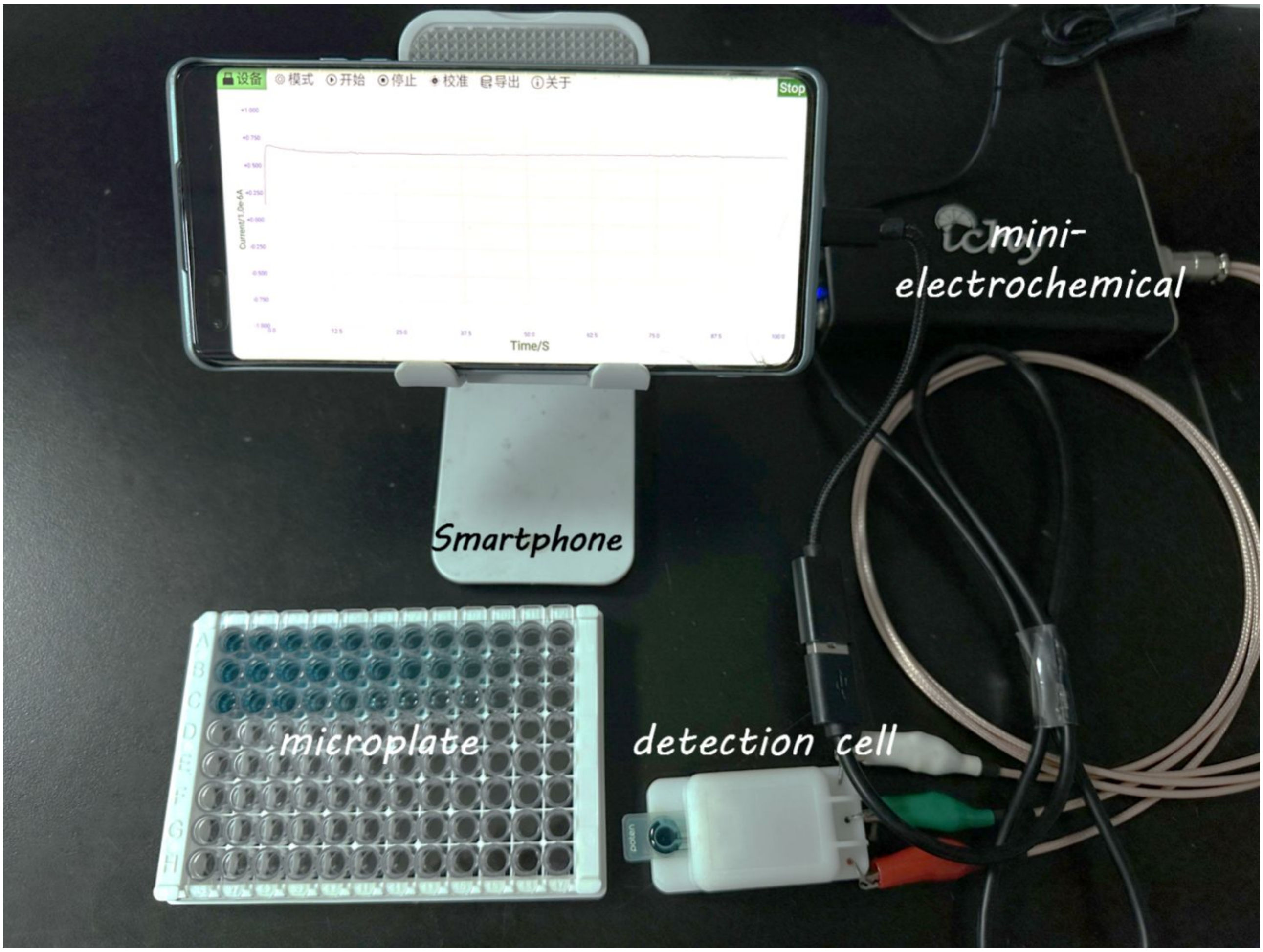

Figure S7. The portable electrochemical detection device.

	1	2	3	4	5	6	7	8	9
T_{surr}/ °C	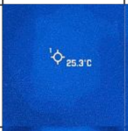	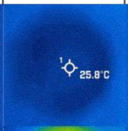	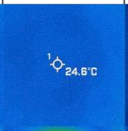	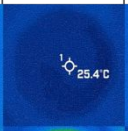	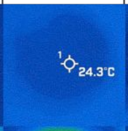	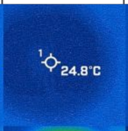	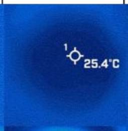	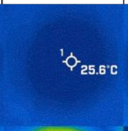	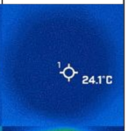
T/ °C	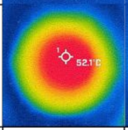	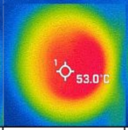	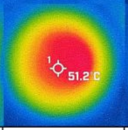	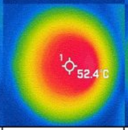	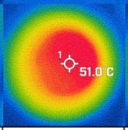	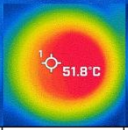	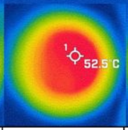	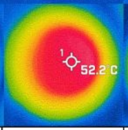	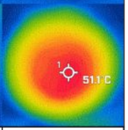
△T/ °C	26.8	27.2	26.6	27.0	26.7	27.6	27.1	26.6	26.0

Figure S8. The stability of thermal images with a mobile phone.

Table S1. Kinetics parameters comparison of the FeMn-NC_{etch}/SAC with other peroxidase-like catalysts.

Nanozymes	K_m / mM		V_{max} / 10^{-8} M s^{-1}		Ref.
	TMB	H_2O_2	TMB	H_2O_2	
HRP	0.434	3.7	10	8.71	1
FeN_5-SA	0.78	24.4	41.029	53.859	2
RF-Fe^{3+}	0.88	0.8	19	18	3
Au-$NPFe_2O_3NC$	0.429	138.5	5.882	4.77	4
Fe_3O_4@MIL-100(Fe) with ATP	0.295	0.294	48.31	7.43	5
FeMn-NC_{etch}/SAC	0.26	0.69	6.3	14.23	This work

Table S2. Comparison of biosensing methods for HER2

Biosensing type[a]	Materials	LOD (ng/mL)	Linear range (ng/mL)	Ref.
EIS biosensor	antibody fragments and gold nanoparticles.	0.01	0.01 - 100	6
Terahertz metasurface biosensor	Aptamer-HB5	0.1	0.1 - 100	7
Photonic crystal fibers	SiO_2@CeO_2	1000	1000 ~	8
Aggregation-induced electrochemiluminescence emitter	L-TPE-TEA	0.0066	0.01 – 500	9
DPV biosensor	PbS QD	0.08	0.1 - 100.0	10
Chronoamperometry immunoassay	FeMn-NC_{etch}/SAC	0.0039	0.01 - 10	This work

[a] EIS: electrochemical impedance spectroscopy employing single-chain fragment variable antibody fragments and gold nanoparticle-modified disposable screen-printed carbon electrodes. DPV: differential pulsed voltammetry.

REFERENCES

(1) Gao, L.; Zhuang, J.; Nie, L.; Zhang, J.; Zhang, Y.; Gu, N.; Wang, T.; Feng, J.; Yang, D.; Perrett, S.; Yan, X. Intrinsic peroxidase-like activity of ferromagnetic nanoparticles. *Nat. Nanotechnol.* **2007**, *2*, 577-583.

(2) Li, R.; Jiao, L.; Jia, X.; Yan, L.; Li, X.; Yan, D.; Zhai, Y.; Zhu, C.; Lu, X. Bioinspired FeN_5 sites with enhanced peroxidase-like activity enable colorimetric sensing of uranyl Ions in seawater. *Anal. Chem.* **2024**, *96*, 3124-3130.

(3) Wang, C.; Zhang, M.; Bai, L.; Gai, P.; Li, F. Light-driven self-cascade peroxidase-like nanozymes without exogenous H_2O_2. *Anal. Chem.* **2023**, *95*, 7014-7020.

(4) Boriachek, K.; Masud, M.; Palma, C.; Phan, H.; Yamauchi, Y.; Hossain, M.; Nguyen, N.; Salomon, C.; Shiddiky, M. Avoiding pre-isolation step in exosome analysis: Direct isolation and sensitive detection of exosomes using gold-loaded nanoporous ferric oxide nanozymes. *Anal. Chem.* **2019**, *91*, 3827-3834.

(5) Xu, J.; Peng, J.; Wang, X.; Hou, X. Enhanced peroxidase-like activity of Fe_3O_4@MIL-100(Fe) aroused by ATP for one-step colorimetric sensing toward cholesterol. *ACS Sustain. Chem. Eng.* **2022**, *10*, 9315-9324.

(6) Sharma, S.; Zapatero-Rodríguez, J.; Saxena, R.; O'Kennedy, R.; Srivastava, S. Ultrasensitive direct impedimetric immunosensor for detection of serum HER2. *Biosens. Bioelectron.* **2018**, *106*, 78-85.

(7) Zeng, Q.; Liu, W.; Lin, S.; Chen, Z.; Zeng, L.; Hu, F. Aptamer HB5 modified terahertz metasurface biosensor used for specific detection of HER2. *Sens. Actuators B* **2022**, *355*, 131337.

(8) Rusyakina, O.; Geernaert, T.; Loyez, M.; Lobry, M.; Chah, K.; Mergo, P.; Thienpont, H.; Caucheteur, C.; Berghmans, F.; Baghdasaryan, T. Cascaded bragg gratings in photonic crystal fiber for plasmonic cladding mode-based biosensing of HER2 protein. *Sens. Actuators B* **2023**, *382*, 133561.

(9) Jia, Y.; Ren, X.; Zhang, X.; Wu, D.; Ma, H.; Li, Y.; Wei, Q. Encapsulation of tetraphenylethylene derivative in liposome vesicles as promising aggregation-induced electrochemiluminescence emitter for detection of human epidermal growth factor receptor 2. *Anal. Chem.* **2023**, *95*, 9139-9144.

(10) Ou, D.; Sun, D.; Lin, X.; Liang, Z.; Zhong, Y.; Chen, Z. A dual-aptamer-based biosensor for specific detection of breast cancer biomarker HER2 via flower-like nanozymes and DNA nanostructures. *J. Mater. Chem. B* **2019**, *7*, 3661-3669.
